# Adverse event reporting of mirtazapine: A disproportionality analysis of FDA adverse event reporting system (FAERS) database from 2004–2024

**DOI:** 10.1371/journal.pone.0328191

**Published:** 2025-12-19

**Authors:** Keying Guo, Haipeng Li, Wei Wang, Ling Cheng, Weina Du, Jing Zhang

**Affiliations:** 1 College of Nursing, Bengbu Medical University, Bengbu, Anhui, People’s Republic of China; 2 College of Mental Health, Bengbu Medical University, Bengbu, Anhui, People’s Republic of China; Northwestern University Feinberg School of Medicine, UNITED STATES OF AMERICA

## Abstract

**Background:**

Mirtazapine is a pharmacological agent commonly utilized as a first-line treatment for major depressive disorder, exhibiting both noradrenergic and selective serotonergic activity. Currently, there is an absence of a comprehensive and systematic review of the adverse events (AEs) associated with mirtazapine. Consequently, this study aims to assess the safety profile of mirtazapine in real-world clinical practice by performing an in-depth analysis of data from the Food and Drug Administration’s Adverse Event Reporting System (FAERS).

**Methods:**

This study collected all real-world adverse event data related to mirtazapine from the FAERS database spanning from Q1 2004 to Q4 2024. Disproportionality analysis methods were employed, including Reporting Odds Ratio (ROR), Proportional Reporting Ratio (PRR), Bayesian Confidence Propagation Neural Network (BCPNN), and Multi-Item Gamma Poisson Shrinker (MGPS), to assess the associations between mirtazapine and major medical events.

**Results:**

Our study identified a total of 14,237 adverse event reports related to mirtazapine from the FAERS database, of which 2,954 had available data. The results identified several previously unreported adverse events, including prolonged QT interval on electrocardiogram, insomnia, coma, falls, and drug toxicity. AEs related to mirtazapine occurred across 27 system organ classes (SOCs), primarily including Nervous System Disorders, Investigations, and Surgical and Medical Procedures. The onset of adverse events typically occurred within one month of mirtazapine administration, though it is important to note that the risk of AEs persists even in the following year.

**Conclusion:**

Our research findings are consistent with clinical observations. Additionally, we have identified new adverse reactions related to mirtazapine. These results provide valuable evidence for further guiding safety research on mirtazapine. They also help clinicians place greater emphasis on monitoring its AEs during use.

## 1. Introduction

Major depressive disorder is a serious psychiatric condition that is strongly linked to an elevated risk of suicidal ideation and behaviors, a marked deterioration in quality of life, and cognitive deficits, resulting in a substantial psychological burden for affected individuals [1]. Mirtazapine, a noradrenergic and specific serotonergic antidepressant, is commonly employed as a first-line pharmacotherapy for the treatment of major depressive disorder [2]. Moreover, mirtazapine is the first antidepressant worldwide to exhibit dual inhibitory effects on norepinephrine (NE) and specific serotonin (5-HT), and is classified as an α-receptor antagonist. Mirtazapine primarily exerts its effects by inhibiting the reuptake of NE and 5-HT, blocking the postsynaptic inhibitory α2 receptors at 5-HT neuronal terminals, thereby enhancing NE and 5-HT activity, which results in sedative effects and effectively alleviates symptoms of anxiety and depression [3]. Additionally, mirtazapine is a potent antagonist of the histamine H1 receptor, which is hypothesized to contribute to its sedative effects and weight gain [4]. Furthermore, in addition to its antidepressant effects, mirtazapine exhibits a range of additional pharmacological actions. By blocking the 5-HT2 receptors, mirtazapine improves sleep quality, helping users achieve better sleep without suppressing rapid eye movement sleep, thereby extending the overall sleep duration [5]. Research has demonstrated that, in comparison with selective serotonin reuptake inhibitors (SSRIs), mirtazapine exhibits a more rapid onset of action and generally does not interfere with sexual function. In patients experiencing sexual dysfunction as a result of SSRIs, transitioning to mirtazapine has been shown to significantly improve sexual function [6]. Terevnikov et al. conducted a randomized controlled trial and found that mirtazapine had a positive effect on cognitive impairment symptoms, without increasing the risk of exacerbating psychiatric disorders [7]. A randomized double-blind controlled trial found that mirtazapine promotes sleep by blocking serotonin and histamine receptors. Compared to a placebo, mirtazapine is more effective in treating insomnia in elderly patients with chronic insomnia [8]. Therefore, mirtazapine is highly effective in treating symptoms of depression and insomnia.

Mirtazapine, a widely utilized pharmacological agent in the treatment of depression, has found extensive clinical application. However, as its use continues to rise, growing concerns regarding its safety profile and potential adverse consequences have prompted heightened attention from both researchers and healthcare professionals. Several studies [9,10] have reported that the most commonly observed AEs associated with mirtazapine include an increased risk of self-harm or suicide, increased appetite and weight gain, dizziness, transient elevations in cholesterol levels and liver function tests, as well as an elevated risk of cardiovascular diseases. Additionally, although rare, there are still studies that have found fatal AEs, such as mirtazapine induced pancreatitis should be considered when patients taking mirtazapine report abdominal discomfort [11]. Therefore, it is crucial to analyze the associated adverse drug events before the clinical application of mirtazapine.

FAERS is a publicly accessible spontaneous reporting system that collects millions of adverse event reports from healthcare professionals, consumers, and other sources. Currently, the FAERS database stands as one of the largest pharmacovigilance databases worldwide, offering a rich resource of data for drug safety research. It has been demonstrated to be highly effective in identifying adverse drug reactions associated with drug exposure [12–14]. However, the FAERS database does have certain limitations, though this does not undermine its continued reliability as a method for analyzing large sample sizes.

Therefore, this study aims to comprehensively describe the safety profile of mirtazapine by analysing post-marketing data from the FAERS database. We employed multiple non-proportionality analysis methods to identify significant adverse event signals associated with mirtazapine use, with particular focus on signals not reported in the product information.

## 2. Materials and methods

### 2.1. Data source and study design

The data for this study was sourced from the FAERS database, a comprehensive pharmacovigilance resource that supports the FDA’s post-market surveillance program for approved drugs and therapeutic biologics. We downloaded all reports from the database covering the period from Q1 2004 to Q4 2024. The FAERS database is categorized into seven distinct sections: DEMO (patient demographics and management information), DRUG (drug-specific details), REAC (encoded adverse events), OUTC (patient outcomes), RPSR (reporting sources), THER (treatment initiation and cessation dates), and INDI (indications for drug use), as well as a category for deleted cases. The FAERS database utilizes the Medical Dictionary for Regulatory Activities (MedDRA) terminology to code AE data. MedDRA categorizes terms into a hierarchical structure consisting of five levels: System Organ Classes (SOCs), High-Level Group Terms (HLGTs), High-Level Terms (HLTs), Preferred Terms (PTs), and Lowest-Level Terms (LLTs) [15]. This study focuses on the System Organ Class (SOC) and Preferred Term (PT) levels to identify potential safety signals more effectively. To address the issue of duplicate reports, we employed the method recommended by the FDA. From the DEMO Table, we extracted the fields PRIMARYID, CASEID, and FDA_DTﬁelds. Following FDA guidance, for each CASEID, we retained the record with the latest FDA_DT. In cases where both CASEID and FDA_DT were identical, the record with the highest PRIMARYID was selected. In the analysis, we extracted all reports where mirtazapine was listed as the “primary suspect” (PS) drug. Reports where mirtazapine was listed as a “concomitant medication” (C), “secondary suspect” (SS), or “interaction” (I) were excluded from the primary analysis to enhance the specificity of signal detection. This exclusion was undertaken because the “primary suspect” designation reflects the original reporter’s strongest causal attribution view.

### 2.2. Statistical analysis

As a widely utilized approach in pharmacovigilance research, disproportionality analysis is employed to detect spontaneous signals, including both frequentist statistics and Bayesian statistics, to comprehensively identify AE signals associated with medications. Frequentist statistics include the Reporting Odds Ratio (ROR) and the Proportional Reporting Ratio (PRR) [16,17], while Bayesian statistics encompass the Multi-item Gamma-Poisson Shrinker (MGPS) and the Bayesian Confidence Propagation Neural Network (BCPNN) [18]. In this study, adverse events that meet the positive threshold criteria of all four methods were classified as AEs. As shown in Table 1, each of the four algorithms was employed to identify at least one positive indicator of drug-related AEs, with the criteria including: a 95% confidence interval (CI) greater than 1, N ≥ 3; PRR ≥ 2, χ2 ≥ 4; IC025 > 0, or EBGM05 > 2. The Tables used in the descriptive analysis are provided in Table 2.

**Table 1:** Four major algorithms used for signal detection.

Algorithms	Equation	Criteria
ROR	ROR = ad/bc	Lower limit of 95%CI > 1, a ≥ 3
	95%CI = eIn(ROR)±1.96(1/a + 1/b + 1/c + 1/d)^0.5	
PRR	PRR = a(c + d)/c/(a + b)	PRR > 2, χ² > 4, a > 3
	χ²=[(ad-bc) ^2](a + b + c + d)/[(a + b)(c + d)(a + c)(b + d)]	
BCPNN	IC = log2a(a + b + c + d)(a + c)(a + b)	IC025 ＞ 0
	95%CI = E(IC)±2V(IC)^0.5	
MGPS	EBGM = a(a + b + c + d)/(a + c)/(a + b)	EBGM05 > 2
	95%CI=[eIn(EBGM±1.96(1/a + 1/b + 1/c + 1/d)^0.5	

Abbreviation: a, number of reports containing both the target drug and target adverse drug reaction; b, number of reports containing other adverse drug reaction of the target drug; c, number of reports containing the target adverse drug reaction of other drugs; d, number of reports containing other drugs and other adverse drug reactions. 95%CI, 95% confidence interval; N, the number of reports; χ2, chi-squared; IC, information component; IC025, the lower limit of 95% CI of the IC; E (IC), the IC expectations; V(IC), the variance of IC; EBGM, empirical Bayesian geometric mean; EBGM05, the lower limit of 95% CI of EBGM.

**Table 2:** Two-by-two contingency Table for disproportionality analyses.

	Target AEs	Other AEs	Total
	a	b	a + b
Other drugs	c	d	c + d
Total	a + c	b + d	a + b + c + d

Abbreviation: AEs, adverse events; a, number of reports containing both the target drug and target adverse drug reaction; b, number of reports containing other adverse drug reaction of the target drug; c, number of reports containing the target adverse drug reaction of other drugs; d, number of reports containing other drugs and other adverse drug reactions.

## 3. Results

### 3.1. General characteristics

**Fig 1:**
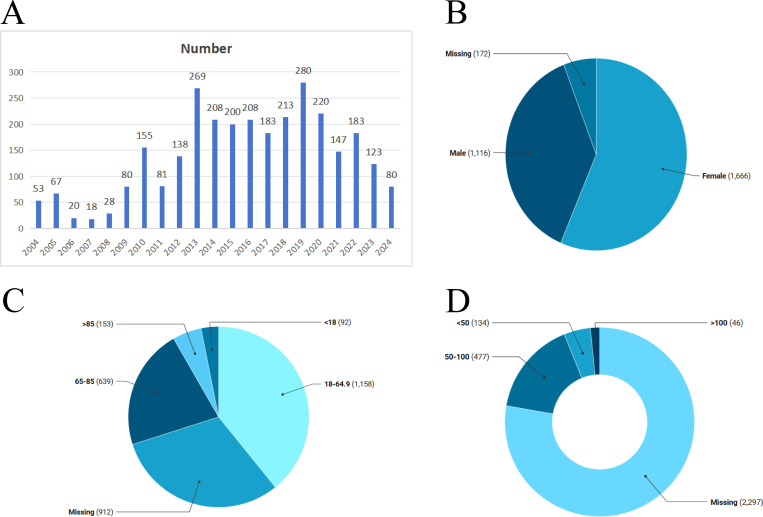
Clinical characteristics of Mirtazapine adverse event reports from the FAERS database.

Between Q1 2004 and Q4 2024, a total of 14,237 AEs reports related to mirtazapine were submitted to the FAERS database, of which 2,954 included complete baseline data. Table 3 presents a detailed description of the characteristics of the mirtazapine-related AEs reports submitted. In all reported cases, female patients represented a higher proportion, accounting for 56.4%. Among the patients, 16.1% had a body weight between 50 and 100 kg, while 1.6% weighed over 100 kg, and 4.5% weighed less than 50 kg. Additionally, 77.8% of the reports lacked weight data. The highest proportion of patients (39.2%) were aged between 18 and 64.9 years, followed by those aged 65–85 years (21.6%) and those over 85 years (5.2%). These distributions are consistent with the epidemiology of the drug’s indications(24). The majority of reports originated from the United States (50.8%), followed by Switzerland (9.9%), France (7.8%), Japan (7.4%), and Canada (5.6%). In terms of report sources, clinicians contributed the highest proportion (34.8%), followed by consumers (32%), other healthcare professionals (15.1%), and pharmacists. The primary outcomes included hospitalization (initial or prolonged) (26.4%), other outcomes (23.1%), and death (10.7%), as shown in Fig 1.

**Table 3:** Clinical characteristics of Mirtazapine adverse event reports from the FAERS database (Q1 2004 – Q4 2024).

Characteristics	Case number (n)	Case proportion (%)
Number of events	2954	
Gender		
Female	1666	56.4%
Male	1116	37.8%
Missing	172	5.8%
Weight (kg)		
<50	134	4.5%
50-100	477	16.1%
>100	46	1.6%
Missing	2297	77.8%
Age (years)		
<18	92	3.1%
18-64.9	1158	39.2%
65-85	639	21.6%
>85	153	5.2%
Missing	912	30.9%
Reporter’s Type of Occupation		
Consumer	944	32.0%
Health professionals	199	6.7%
Lawyer	12	0.4%
Medical doctor	1029	34.8%
Pharmacist	203	6.9%
Other health-professional	447	15.1%
Missing	120	4.1%
Reporting countries (Top 5)		
United States	1500	50.8%
Switzerland	291	9.9%
France	230	7.8%
Japan	220	7.4%
Canada	166	5.6%
Outcome		
Congenital anomaly	11	0.4%
Death	316	10.7%
Disability	62	2.1%
Hospitalization	779	26.4%
Life-threatening	127	4.3%
Other	682	23.1%
Required intervention to prevent permanent impairment/damage	9	0.3%
Missing	968	32.8%

### 3.2. Signal detection of Mirtazapine at the system organ class (SOC) level

**Fig 2:**
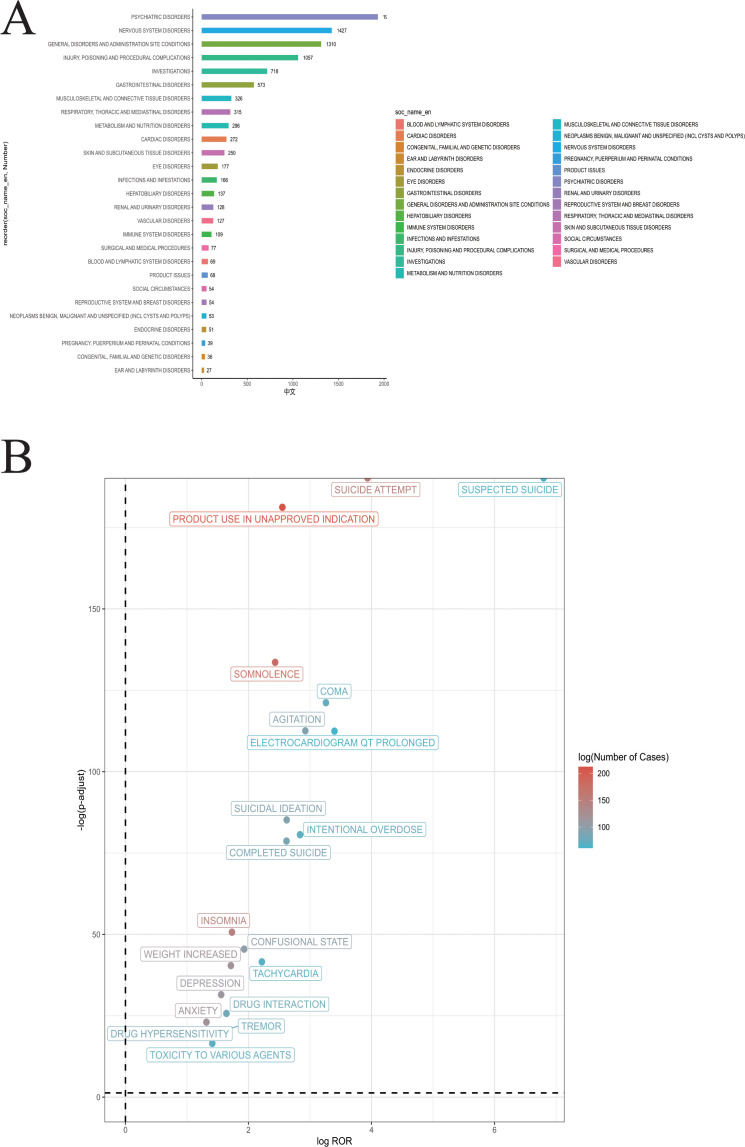
Fig 2A Signal Strength of top20 AEs of Mirtazapine at the System Organ Class (SOC) Level in FAERS Database. Fig 2B volcano plot of the top 20 PT signals by occurrence in the FAERS database.

The AE reports for Mirtazapine at the SOC level are presented in Table 4. The distribution of SOCs in reports of Mirtazapine-related AEs is shown in Fig 2A. The results indicate that the most prominent signal was observed in Nervous system disorders (n = 1427, ROR 1.82 [1.72–1.93], PRR 1.7 [451.03], EBGM 1.7 [1.62], IC 0.77 [0.68]), Investigations (n = 718, ROR 1.19 [1.1–1.28], PRR 1.17 [19.26], EBGM 1.17 [1.1], IC 0.23 [0.12]), and Surgical and medical procedures (n = 77, ROR 0.57 [0.45–0.71], PRR 0.57 [25.35], EBGM 0.57 [0.47], IC −0.81 [−1.14]). Nervous system disorders (n = 1427, ROR 1.82 [1.72–1.93], PRR 1.7 [451.03], EBGM 1.7 [1.62], IC 0.77 [0.68]) exhibited strong positive signals across all four algorithms.

**Table 4:** Signal strength of Mirtazapine AEs across System Organ Classes (SOC) in the FAERS database.

System Organ Class (SOC)	Case numbers	ROR (95%Cl)	PRR (χ^2^)	EBGM (EBGM05)	IC (IC025)
Nervous system disorders	1427	1.82 (1.72 - 1.93)	1.7 (451.03)	1.7 (1.62)	0.77 (0.68)
Investigations	718	1.19 (1.1 - 1.28)	1.17 (19.26)	1.17 (1.1)	0.23 (0.12)
Surgical and medical procedures*	77	0.57 (0.45 - 0.71)	0.57 (25.35)	0.57 (0.47)	−0.81 (−1.14)
Vascular disorders	127	0.59 (0.49 - 0.7)	0.59 (35.98)	0.59 (0.51)	−0.75 (−1.01)
Injury, poisoning and procedural complications*	1057	1.14 (1.07 - 1.22)	1.13 (16.57)	1.13 (1.07)	0.17 (0.08)
Psychiatric disorders	1932	4.03 (3.83 - 4.23)	3.43 (3534.47)	3.43 (3.29)	1.78 (1.71)
Cardiac disorders	272	1.04 (0.92 - 1.17)	1.04 (0.44)	1.04 (0.94)	0.06 (−0.12)
General disorders and administration site conditions*	1310	0.72 (0.68 - 0.76)	0.76 (123.58)	0.76 (0.72)	−0.4 (−0.49)
Gastrointestinal disorders	573	0.66 (0.6 - 0.71)	0.68 (97.44)	0.68 (0.63)	−0.57 (−0.69)
Endocrine disorders	51	2.02 (1.53 - 2.66)	2.01 (26.08)	2.01 (1.6)	1.01 (0.61)
Musculoskeletal and connective tissue Disorders	326	0.61 (0.55 - 0.68)	0.62 (79.07)	0.62 (0.57)	−0.68 (−0.85)
Eye disorders	177	0.89 (0.76 - 1.03)	0.89 (2.56)	0.89 (0.78)	−0.17 (−0.39)
Metabolism and nutrition disorders	296	1.4 (1.25 - 1.57)	1.39 (33.03)	1.39 (1.26)	0.47 (0.3)
Respiratory, thoracic and mediastinal disorders	315	0.65 (0.58 - 0.73)	0.66 (55.93)	0.66 (0.61)	−0.59 (−0.75)
Social circumstances	54	1.25 (0.96 - 1.64)	1.25 (2.73)	1.25 (1)	0.32 (−0.07)
Skin and subcutaneous tissue disorders	250	0.45 (0.4 - 0.51)	0.47 (160.73)	0.47 (0.42)	−1.1 (−1.28)
Infections and infestations	166	0.3 (0.26 - 0.36)	0.32 (259.04)	0.32 (0.28)	−1.66 (−1.89)
Ear and labyrinth disorders	27	0.63 (0.43 - 0.92)	0.63 (5.78)	0.63 (0.46)	−0.66 (−1.21)
Renal and urinary disorders	128	0.7 (0.59 - 0.83)	0.7 (16.53)	0.7 (0.61)	−0.51 (−0.77)
Neoplasms benign, malignant and unspecified (incl cysts and polyps)*	53	0.2 (0.15 - 0.26)	0.2 (171.42)	0.2 (0.16)	−2.31 (−2.7)
Immune system disorders	109	0.98 (0.82 - 1.19)	0.99 (0.03)	0.99 (0.84)	−0.02 (−0.3)
Hepatobiliary disorders*	137	1.52 (1.28 - 1.8)	1.51 (23.94)	1.51 (1.31)	0.6 (0.35)
Product issues*	68	0.43 (0.34 - 0.54)	0.43 (51.66)	0.43 (0.35)	−1.21 (−1.56)
Pregnancy, puerperium and perinatal conditions*	39	0.92 (0.67 - 1.26)	0.92 (0.27)	0.92 (0.71)	−0.12 (−0.58)
Congenital, familial and genetic disorder*	36	1.19 (0.86 - 1.65)	1.19 (1.1)	1.19 (0.91)	0.25 (−0.22)
Blood and lymphatic System disorders	69	0.4 (0.32 - 0.51)	0.41 (60.4)	0.41 (0.33)	−1.29 (−1.64)
reproductive system and breast disorders	54	0.66 (0.51 - 0.87)	0.67 (9.12)	0.67 (0.53)	−0.59 (−0.98)

Abbreviation: Asterisks (*) indicate statistically significant signals in four algorithms; ROR, reporting odds ratio; PRR, proportional reporting ratio; EBGM, empirical Bayesian geometric mean; EBGM05, the lower limit of the 95% CI of EBGM; IC, information component; IC025, the lower limit of the 95% CI of the IC; CI, confidence interval; AEs, adverse events.

### 3.3. Signal detection of Mirtazapine at the preferred terms (PT) level

In this analysis, the volcano plot results show that the most common AE occurred in Suicide attempt (see Fig 2B). Table 5 provides a detailed list of the top 20 PTs, with the three most frequent being Product use in unapproved indication (n = 212), Drug ineffective (n = 200), and Somnolence (n = 175). Four computational algorithms were applied to assess adverse drug reactions at the PT level, using predefined screening thresholds. Among these, 167 PTs fulfilled the criteria set by all four algorithms. The top 20 AEs associated with Mirtazapine were identified and presented according to the strength of the signal sources for those PTs that satisfied the criteria of all four algorithms (see Table 6). The results indicate that the use of the product in unapproved indications (n = 64, ROR 5.86 [5.12–6.72], PRR 5.76 [835.94], EBGM 5.75 [5.13], IC 2.52 [2.32]), somnolence (n = 175, ROR 5.4 [4.65–6.27], PRR 5.32 [615.85], EBGM 5.32 [4.69], IC 2.41 [2.19]), and suicide attempts (n = 150, ROR 15.3 [13.02–17.98], PRR 15.08 [1968.5], EBGM 15.04 [13.14], IC 3.91 [3.67]) were identified as high-frequency signals. Additionally, several noteworthy AEs not included in the drug label, such as electrocardiogram QT prolonged, insomnia, coma, falls, drug ineffective and drug interaction, were identified among the top 20 most common AEs. These findings may provide valuable insights for updating the AEs section of the mirtazapine prescribing information.

**Table 5:** Top 20 frequency of adverse events at the PT level for Mirtazapine.

PT	Case numbers	ROR (95%Cl)	PRR (χ2)	EBGM (EBGM05)	IC (IC025)
Product use in unapproved indication*	212	5.86 (5.12 - 6.72)	5.76 (835.94)	5.75 (5.13)	2.52 (2.32)
Drug ineffective*	200	0.93 (0.81 - 1.07)	0.93 (1.14)	0.93 (0.83)	−0.11 (−0.31)
Somnolence	175	5.4 (4.65 - 6.27)	5.32 (615.85)	5.32 (4.69)	2.41 (2.19)
Suicide attempt*	150	15.3 (13.02 - 17.98)	15.08 (1968.5)	15.04 (13.14)	3.91 (3.67)
Insomnia*	145	3.32 (2.81 - 3.91)	3.28 (230.93)	3.28 (2.86)	1.71 (1.47)
Weight increased	117	3.28 (2.74 - 3.94)	3.26 (183.42)	3.25 (2.79)	1.7 (1.44)
Anxiety	117	2.49 (2.07 - 2.98)	2.47 (102.76)	2.47 (2.12)	1.3 (1.04)
Depression	112	2.94 (2.44 - 3.55)	2.92 (142.08)	2.92 (2.5)	1.55 (1.27)
Confusional state	101	3.81 (3.14 - 4.64)	3.79 (207.44)	3.78 (3.21)	1.92 (1.63)
Nausea	96	0.75 (0.61 - 0.91)	0.75 (8.2)	0.75 (0.63)	−0.42 (−0.71)
Dizziness	93	1.14 (0.93 - 1.4)	1.14 (1.64)	1.14 (0.96)	0.19 (−0.11)
Suicidal ideation	92	6.15 (5.01 - 7.55)	6.1 (392.74)	6.1 (5.13)	2.61 (2.31)
Agitation	92	7.59 (6.18 - 9.32)	7.53 (520.47)	7.52 (6.33)	2.91 (2.61)
Fatigue	90	0.71 (0.58 - 0.87)	0.71 (10.71)	0.71 (0.6)	−0.49 (−0.8)
Fall*	88	1.62 (1.31 - 1.99)	1.61 (20.49)	1.61 (1.35)	0.69 (0.38)
Completed suicide	85	6.14 (4.96 - 7.61)	6.1 (362.45)	6.09 (5.1)	2.61 (2.29)
Drug hypersensitivity*	84	2.59 (2.09 - 3.21)	2.58 (81.32)	2.58 (2.15)	1.37 (1.05)
Off label use	83	0.61 (0.49 - 0.76)	0.61 (20.58)	0.61 (0.51)	−0.71 (−1.02)
Drug interaction	81	3.12 (2.51 - 3.88)	3.1 (115.72)	3.1 (2.58)	1.63 (1.31)
Tremor	77	2.81 (2.25 - 3.52)	2.8 (89.15)	2.8 (2.32)	1.48 (1.16)

Abbreviation: Asterisks (*) indicate statistically significant signals in four algorithms; ROR, reporting odds ratio; PRR, proportional reporting ratio; EBGM, empirical Bayesian geometric mean; EBGM05, the lower limit of the 95% CI of EBGM; IC, information component; IC025, the lower limit of the 95% CI of the IC; CI, confidence interval; AEs, adverse events.

**Table 6:** Signal strength of top 20 AEs of Mirtazapine at the preferred terms level in FAERS database.

PT	Case numbers	ROR (95%Cl)	PRR (χ2)	EBGM (EBGM05)	IC (IC025)
Product use in unapproved indication*	212	5.86 (5.12 - 6.72)	5.76 (835.94)	5.75 (5.13)	2.52 (2.32)
Somnolence	175	5.4 (4.65 - 6.27)	5.32 (615.85)	5.32 (4.69)	2.41 (2.19)
Suicide attempt	150	15.3 (13.02 - 17.98)	15.08 (1968.5)	15.04 (13.14)	3.91 (3.67)
Insomnia*	145	3.32 (2.81 - 3.91)	3.28 (230.93)	3.28 (2.86)	1.71 (1.47)
Weight increased	117	3.28 (2.74 - 3.94)	3.26 (183.42)	3.25 (2.79)	1.7 (1.44)
Anxiety	117	2.49 (2.07 - 2.98)	2.47 (102.76)	2.47 (2.12)	1.3 (1.04)
Depression	112	2.94 (2.44 - 3.55)	2.92 (142.08)	2.92 (2.5)	1.55 (1.27)
Confusional state	101	3.81 (3.14 - 4.64)	3.79 (207.44)	3.78 (3.21)	1.92 (1.63)
Suicidal ideation	92	6.15 (5.01 - 7.55)	6.1 (392.74)	6.1 (5.13)	2.61 (2.31)
Agitation	92	7.59 (6.18 - 9.32)	7.53 (520.47)	7.52 (6.33)	2.91 (2.61)
Completed suicide	85	6.14 (4.96 - 7.61)	6.1 (362.45)	6.09 (5.1)	2.61 (2.29)
Drug hypersensitivity*	84	2.59 (2.09 - 3.21)	2.58 (81.32)	2.58 (2.15)	1.37 (1.05)
Drug interaction*	81	3.12 (2.51 - 3.88)	3.1 (115.72)	3.1 (2.58)	1.63 (1.31)
Tremor	77	2.81 (2.25 - 3.52)	2.8 (89.15)	2.8 (2.32)	1.48 (1.16)
Coma*	74	9.57 (7.61 - 12.03)	9.5 (562.51)	9.49 (7.83)	3.25 (2.91)
Intentional overdose*	71	7.15 (5.66 - 9.04)	7.11 (372.59)	7.1 (5.84)	2.83 (2.49)
Toxicity to various agents	71	2.66 (2.11 - 3.36)	2.65 (72.92)	2.65 (2.18)	1.4 (1.06)
Tachycardia	67	4.65 (3.65 - 5.91)	4.62 (190.18)	4.62 (3.78)	2.21 (1.86)
Suspected suicide	64	111.27 (86.81-142.62)	110.55 (6811.34)	108.39 (88.06)	6.76 (6.4)
Electrocardiogram qt prolonged*	61	10.54 (8.2 - 13.56)	10.48 (522.68)	10.47 (8.48)	3.39 (3.02)

Abbreviation: Asterisks (*) indicate statistically significant signals in four algorithms; ROR, reporting odds ratio; PRR, proportional reporting ratio; EBGM, empirical Bayesian geometric mean; EBGM05, the lower limit of the 95% CI of EBGM; IC, information component; IC025, the lower limit of the 95% CI of the IC; CI, confidence interval; AEs, adverse events.

### 3.4. Time-to-onset analysis

**Fig 3:**
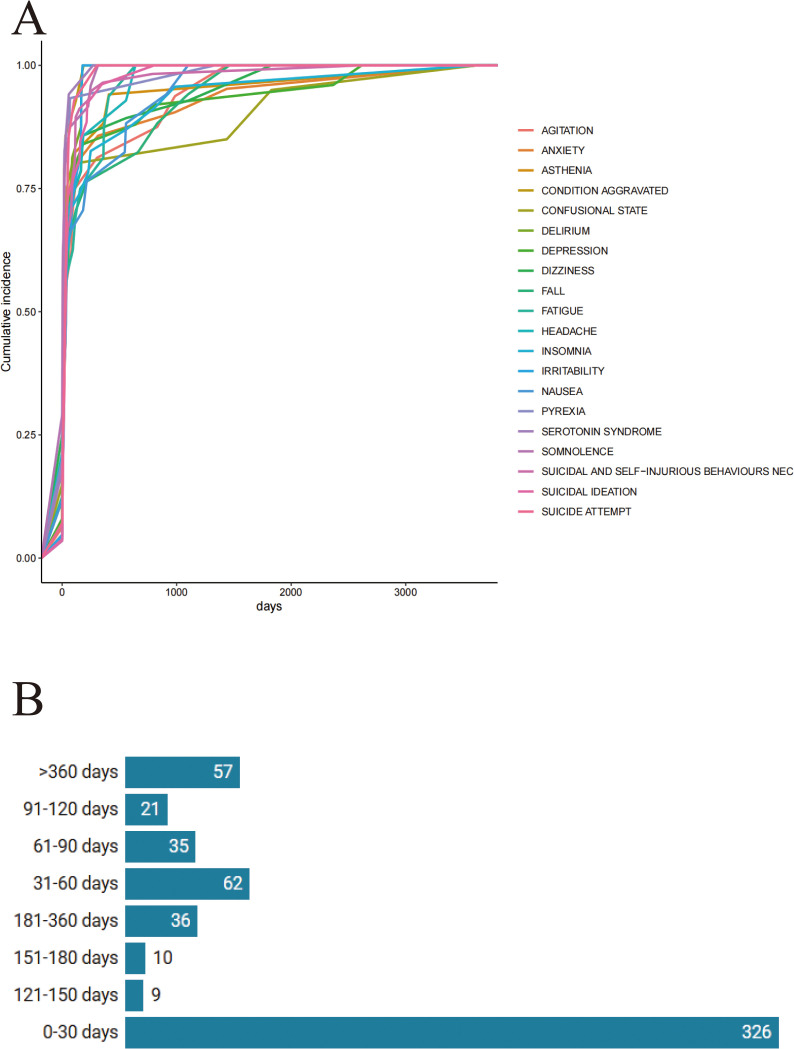
Fig 3A Cumulative incidence of adverse events mirtazapine-related over time. Fig 3B Time to onset of mirtazapine-related adverse events.

The occurrence times of AEs with mirtazapine vary, with a notably broad time span for condition aggravation, as shown in Fig 3A. The majority of AEs related to mirtazapine occur within the first month of use. However, it is important to note that there remains a risk of AEs even one year after starting the medication. The distribution of AEs onset times is illustrated in Fig 3B.

## 4. Discussion

This study, leveraging data from the FAERS database, undertook an extensive pharmacovigilance analysis of the AEs associated with Mirtazapine, offering unparalleled precision and depth in the characterization of these events. It should be noted that conducting reliable and conclusive dose-response analyses based on data from voluntary reporting systems such as FAERS presents significant methodological challenges. Preliminary exploratory analyses have only revealed aggregate reporting outcomes for adverse events, and their results should be interpreted with caution.

In our study, a total of 2,954 data points with complete baseline information and associated with Mirtazapine were identified. The results revealed a higher incidence of AEs in females (56.4%) compared to males (37.8%), indicating a gender susceptibility to AEs. The observed phenomenon may stem from the fact that mirtazapine primarily exerts therapeutic effects on patients with depression, while women exhibit heightened susceptibility to depression, predominantly owing to increased vulnerability associated with hormonal fluctuations [19]. Our findings should be interpreted in the context of the database’s geographical composition, wherein reports from the United States constituted the majority (50.8%). This overrepresentation may introduce bias, as prescribing practices, patient demographics, and adverse event reporting behaviors differ across regions. Consequently, the generalizability of our identified safety signals to other populations, particularly in non-Western countries, may be limited and warrants further investigation. Some reports originate from non-medical professionals, and such reports may exhibit limitations in the technical accuracy of symptom descriptions and the precision of causal attributions. Although they constitute a relatively small proportion, they may nonetheless influence the strength of signals for specific types of adverse events. Regarding body weight, due to the absence of weight data in 77.8% of the reports, this study did not examine the potential impact of body weight on the incidence of AEs. Individuals aged between 18 and 64.9 years comprise 39.2% of the cases and are more prone to experiencing AEs. We attribute this phenomenon to the higher levels of life stress commonly encountered in this age group, which may contribute to disruptions in mental and sleep states. The primary sources of reports are clinical physicians and consumers, as the FAERS database operates as a self-reporting system, with most reports submitted by physicians [20]. The findings indicate that inpatient treatment constitutes the largest proportion, possibly due to the occurrence of more severe conditions that necessitate hospitalization for closer monitoring and to prevent exacerbation of the condition [21]. It must be emphasised that elderly patients, owing to their unique pharmacokinetic characteristics, high prevalence of comorbidities and polypharmacy, are indeed a high-risk group for adverse drug reactions. As a comprehensive assessment of the overall safety profile of mirtazapine, this study primarily aimed to identify safety signals across the entire population. Nevertheless, we recognise the necessity for in-depth investigations into specific high-risk groups. Consequently, conducting dedicated analyses on key populations such as the elderly holds significant clinical importance. Our team will therefore deepen our exploration of the unique risk-benefit characteristics observed in this demographic.

Our study revealed that at the SOC level, nervous system disorders were the most frequent AE, which included emotional symptoms such as anxiety, agitation, and irritability. Anxiety and emotional agitation are common AEs related to the psychiatric side effects of Mirtazapine. A study by Biswas et al. reported cases of exacerbation of anxiety and agitation among individuals taking Mirtazapine [22]. We believe that the AEs induced by Mirtazapine are related to its pharmacological mechanism. Mirtazapine can block the α2-adrenergic heteroreceptors on serotonergic neurons in the dorsal raphe and the presynaptic α2-adrenergic autoreceptors in the locus coeruleus, primarily resulting in increased release of 5-HT and NE [23]. To a certain extent, this may exacerbate anxiety and emotional agitation among individuals undergoing medication. Hence, we recommend performing a mental status evaluation both at the initiation of treatment and continuously throughout the treatment process, alongside closely monitoring the medication dosage. In cases of patients exhibiting mental abnormalities, prompt psychological counseling should be provided, which may help mitigate the occurrence of AEs to some extent.

Our study further reveals that “Product use in unapproved indication,” “Drug ineffectiveness,” and “Somnolence” are also common AEs associated with Mirtazapine. The occurrence of the “Product use in unapproved indication” AE indicates the improper use of Mirtazapine. This suggests that the improper usage may be attributed to either a lack of experience with Mirtazapine among physicians or poor patient adherence to medication [24]. Therefore, further education on the indications and contraindications of Mirtazapine should be provided to clinicians, and better communication with patients regarding the correct, regular, and adequate use of the medication should be established to improve patient adherence. The term “Drug ineffective” is not mentioned on the label. We hypothesize that the outcome may be significantly related to the nature and progression of the indications in the patient population receiving this treatment [25]. Regarding somnolence, it is frequently mentioned as a common AE in patients receiving mirtazapine treatment. For instance, in a randomized controlled trial by Hunter, a subset of patients was unable to tolerate mirtazapine and discontinued the medication due to somnolence or hallucinations [26]. Beatrice et al. conducted a study and identified that the most commonly observed AEs associated with the administration of Mirtazapine include sedation [27]. This is due to the distinct mechanism of action of mirtazapine compared to other antidepressants. Mirtazapine effectively antagonizes 5HT-2, 5HT-3, and H1 receptors, and through this inhibition, it prevents excessive excitation of serotonergic neurons. However, this action may lead to an overly sedative effect, resulting in somnolence [28,29]. This finding is consistent with the known risks indicated in the drug label, further confirming the reliability of the data. Therefore, to avoid these AEs, it is important to avoid excessive dosing, as higher doses prescribed more frequently are associated with a greater incidence of AEs.

This study also identified AEs associated with suicide attempts. Our findings are consistent with those of previous studies, which have shown that patients taking mirtazapine have a higher suicide risk compared to those taking other antidepressants (e.g., SSRIs, zolpidem, etc.) [30,31]. Furthermore, suicide events occurred among adult trial participants during the clinical trial phase prior to the release of mirtazapine. This phenomenon is attributed to mirtazapine’s dual mechanism of action, which differs from other antidepressants. It does not inhibit the reuptake of serotonin and catecholamines, but rather enhances the release of norepinephrine, particularly serotonin transmission mediated by 5-HT1A. This receptor-mediated mechanism of action may lead to unsTable mood fluctuations, which is why some patients may exhibit suicidal tendencies and other adverse behaviors [32]. Another possibility is that patients develop tolerance to mirtazapine, and the effectiveness of these drugs often diminishes over time, leading to a worsening of depressive symptoms and the emergence of suicidal thoughts [33]. It should be emphasised that the interpretation of the significant signals associated with suicidal tendencies detected in this study requires particular caution. This finding is likely to be subject to substantial “indication confounding” bias. Specifically, mirtazapine is primarily prescribed for the treatment of major depressive disorder, a condition itself associated with a markedly elevated risk of suicide. Consequently, the observed signal may reflect the underlying disease characteristics of the patient population receiving this medication rather than a direct causal effect of the drug itself. Also, Kamp et al. concluded that insufficient data are available to determine the impact of mirtazapine on the risk of suicide, suicide attempts, and serious AEs. The discrepancies in these study results may stem from differences in the study populations included, or from biases such as selection bias, information bias, or observer bias, which may not have been adequately addressed and could influence the findings [28]. Therefore, it is recommended to conduct randomized controlled trials in the future to investigate the suicide-related AEs associated with mirtazapine, in order to better assess the drug’s benefits and risks and assist clinicians in mitigating potential mirtazapine-induced AEs. Besides, our study compared mirtazapine with other antidepressants and found that certain adverse reactions are common among antidepressants, such as weight gain, ECG abnormalities, and the emergence of suicidal ideation. However, particular attention should be paid to the higher risk of suicidal ideation in patients using mirtazapine, primarily due to its dual mechanism of action distinct from other antidepressants [34]. Post-SSRI sexual dysfunction has recently been identified as a potential adverse effect of SSRIs and SNRIs. However, our analysis showed a weaker signal association between mirtazapine and sexual dysfunction. This aligns with previous clinical observations that mirtazapine, due to its antagonism of the 5-HT2C receptor, has a lesser impact on sexual function and is sometimes even used to treat sexual dysfunction induced by SSRIs. By comparing it with other antidepressant classes, our study reinforces the notion that mirtazapine possesses a unique safety profile. Physicians can personalize the selection of mirtazapine based on individual patient circumstances, such as those presenting with symptoms of anorexia or sexual dysfunction.

Moreover, it is noteworthy that this study identified an unmentioned AEs related to prolonged Electrocardiogram QT at the PT level. This finding is consistent with the results of Matsuda et al., which showed that even at low doses of mirtazapine, patients experienced significant QTc interval prolongation [35]. Furthermore, a study by Natalia et al. found that compared to non-selective serotonin reuptake inhibitors, mirtazapine was associated with a higher risk of sudden cardiac death and ventricular arrhythmias [36]. Mirtazapine may cause prolonged QT intervals on the electrocardiogram, potentially due to its electrophysiological effects on ion channels. These effects can influence the cardiac action potential, prolong depolarization and repolarization phases, widen the QRS complex, and thereby extend the QT interval or induce a Brugada-like ECG pattern [37]. To prevent such events, healthcare professionals should regularly monitor the electrocardiogram both before starting treatment and during the treatment process, particularly during the initial phase and dose adjustments, to ensure the QT interval remains within a safe range.

In addition to the newly identified AEs mentioned above, this study also discovered other associated AEs, including tremors, weight increase, fatigue, dizziness, nausea, and tachycardia. These AEs were detected with corresponding signals in this study, and the reliability of the data was subsequently validated. Our analysis also identified several novel and unforeseen AEs that were not previously addressed in the documentation or regulatory trials, including insomnia, coma, falls, drug ineffective and drug interaction. But, the precise mechanisms underlying these AEs remain unclear. However, these findings highlight the necessity of continuous monitoring and reporting of AEs, particularly those that were not anticipated in the initial clinical trials [38]. In clinical practice, healthcare professionals should accurately assess patients’ risk factors, inform them of potential AEs, and conduct regular follow-up monitoring of their health. This helps healthcare providers promptly identify and manage drug-related AEs, thereby enhancing medication safety for patients.

The study also revealed that the majority of AEs occurred during the first month of mirtazapine administration. However, after one year of administration, the incidence of AEs increased once again. The differences in the timing of AEs across different classes of drugs reflect the metabolic processes of the drugs within the body and their long-term or short-term effects on various physiological systems [39]. Suicidal behavior, anxiety, hypersomnia, and other psychiatric abnormalities typically require more stringent monitoring in the later stages of treatment to prevent chronic toxicity or cumulative effects. Therefore, future clinical studies should extend the follow-up period and closely observe the symptoms of patients during periods when AEs are more likely to occur, to provide a more comprehensive assessment of the AEs of mirtazapine.

Our study acknowledges several limitations. First, the FAERS database may introduce potential bias due to the voluntary nature of the data submission by pharmaceutical companies, healthcare professionals, and consumers. Notably, reports from the general public may be prone to inaccuracies or incompleteness, which could subsequently impact the validity of our analysis. Additionally, the majority of FAERS reports originate from the United States, which, due to variations in drug use across countries and ethnic differences, limits the external validity of the findings to other populations. Despite these limitations, our findings still provide valuable guidance for healthcare professionals, facilitating detailed follow-up and monitoring of mirtazapine-related AEs. Therefore, future research could include prospective clinical studies in conjunction with epidemiological research to achieve a more accurate assessment of mirtazapine.

## 5. Conclusion

This study evaluated the safety of mirtazapine using the FAERS database. Mirtazapine’s most common AEs include suicide attempt, insomnia, anxiety, fatigue, tremors, weight increase, dizziness, nausea, and tachycardia. In addition to these, electrocardiogram QT prolonged, insomnia, coma, falls, drug ineffective and drug interaction have not been previously reported. These findings underscore the importance for clinicians to remain vigilant regarding novel and unforeseen adverse reactions, facilitating their early identification and the timely implementation of appropriate interventions.

## Supporting information

S1 Data Raw data. (CSV)
